# Supplementary material for: Predictors of post-stroke cognitive impairment at three-month following first episode of stroke among patients attended at tertiary hospitals in Dodoma, central Tanzania: A protocol of a prospective longitudinal observational study metadata
Source: PLoS One. 2023 Mar 2;18(3):e0273200. doi: 10.1371/journal.pone.0273200 (PMC9980770; doi:10.1371/journal.pone.0273200)
Supplement: S1 File — (PDF) [file pone.0273200.s001.pdf]

THE UNIVERSITY OF DODOMA

OFFICE OF THE DEPUTY VICE CHANCELLOR-ARC

DIRECTORATE OF RESEARCH, PUBLICATIONS AND CONSULTANCY
P.O. Box 259
DODOMA, TANZANIA
TEL: +255-026-2310002 FAX: +255-026-2310012
EMAIL: dvcarc@udom.ac.tz;
Website address:www.udom.ac.tz

Ref. No. MA.84/261/02/ 18th June, 2021

To: Baraka O. Alphonce
The University of Dodoma

RE: REQUEST FOR ETHICAL CLEARANCE

The heading above is concerned

The Institutional Research Review Ethics Committee (IRREC) hold its 50th on 17th May 2021 and reviewed research proposal titled "***Prevalence Clinical pattern and Predictors of post stroke Cognitive Impairment among Adult Patient with First stroke Admitted at Tertiary Hospitals Dodoma.***" I am glad to inform you that the committee has granted ethical clearance on the submitted research proposal.

Furthermore, as the Principal Investigator of the study, the following conditions must be fulfilled:

- Progress report is submitted to the University of Dodoma.
- Copies of final publications are made available to the University of Dodoma.
- Sites: **Dodoma Region.**

Best regards,

Dr. Alex Mongi
Chairperson - Institutional Research Review Committee (IRREC)

C: C: Deputy Vice Chancellor-Academic, Research and Consultancy
